# Efficient Sequential Detection of Two Antibiotics Using a Fiber-Optic Surface Plasmon Resonance Sensor

**DOI:** 10.3390/s24072126

**Published:** 2024-03-26

**Authors:** Ze Zhao, Huiting Yin, Jingzhe Xiao, Mei Cui, Renliang Huang, Rongxin Su

**Affiliations:** 1 State Key Laboratory of Chemical Engineering, Tianjin Key Laboratory of Membrane Science and Desalination Technology, School of Chemical Engineering and Technology, Tianjin University, Tianjin 300072, China; zhao_ze@tju.edu.cn (Z.Z.);; 2 Zhejiang Institute of Tianjin University, Ningbo 315201, China; yinht97@tju.edu.cn; 3 Tianjin Key Laboratory for Marine Environmental Research and Service, School of Marine Science and Technology, Tianjin University, Tianjin 300072, China

**Keywords:** antibiotics, fiber-optic SPR, surface plasmon resonance, antibody

## Abstract

Antibiotic residues have become a worldwide public safety issue. It is vital to detect multiple antibiotics simultaneously using sensors. A new and efficient method is proposed for the combined detection of two antibiotics (enrofloxacin (Enro) and ciprofloxacin (Cip)) in milk using surface plasmon resonance (SPR) sensors. Based on the principle of immunosuppression, two antibiotic antigens (for Enro and Cip) were immobilized on an optical fiber surface with conjugates of bovine serum albumin using dopamine (DA) polymerization. Each single antigen was bound to its corresponding antibody to derive standard curves for Enro and Cip. The fiber-optic sensor’s sensitivity was 2900 nm/RIU. Detection limits were calculated to be 1.20 ng/mL for Enro and 0.81 ng/mL for Cip. The actual system’s recovery rate was obtained by testing Enro and Cip in milk samples; enrofloxacin’s and ciprofloxacin’s mean recoveries from the milk samples were 96.46–120.46% and 96.74–126.9%, respectively. In addition, several different regeneration solutions were tested to analyze the two target analytes’ regeneration ability; NaOH and Gly-HCl solutions were found to have the best regeneration ability.

## 1. Introduction

Antibiotic residues have attracted widespread attention due to their harmful effects on the environment, ecology, and human health [1]. Fluoroquinolone antibiotics are widely used in animal husbandry and aquaculture because of their long usage duration, wide antibacterial spectrum, and strong antibacterial activity [2,3,4]. In recent years, fluoroquinolones have received significant attention [5]. The overuse of fluoroquinolones has significantly exacerbated the spread of antibiotic resistance, which has been recognized as a major global problem that must be addressed [6,7]. In addition, the widespread use of fluoroquinolones in animal agriculture is leading to the accumulation of drug residues in the food chain and in water sources [8]. Many countries have now established maximum residue limits (MRLs) for antibiotics in foods of animal origin. However, there are many types of antibiotics in foods, and most antibiotic residues in complicated samples are measured in the PPM range. Therefore, the detection of trace antibiotics in these complex substrates is urgently required [9,10].

Biorecognition molecules such as antibodies and enzymes are highly specific in practical detection processes, and are usually used for analytical and diagnostic purposes [11,12]. When biorecognition molecules are used as biosensors they have highly sensitive detection capacities [13,14]. However, producing these biometric molecules is costly and time-consuming, and the production process is unstable, resulting in variations across different production batches. They are also more sensitive to factors such as ambient temperature and pH [15,16]. Therefore, there is an urgent need to develop a more stable and reusable biorecognition molecule for detecting antibiotics [17].

Surface plasmon resonance (SPR) sensing technology offers real-time monitoring, label-free sensing, and high sensitivity [18,19,20,21]. It can be used in environmental monitoring [22], new drug development [23], food science [24], and biology [25]. SPR sensors are sensitive to refractive index changes on surfaces, which allows the dynamic monitoring of biomolecular interactions [26,27,28,29,30]. SPR sensors can be broadly classified as fiber-optic, prism-coupled, grating, and integrated optical waveguide types. Among these, fiber-optic SPR sensors have attracted significant attention due to their miniaturization and portability [31]. Although some conventional analytical techniques, including high-performance liquid chromatography (HPLC) [32], mass spectrometry (MS) [33], ELISA [34], SERS [35], etc., have been developed and applied to analyze substances’ compositions, these techniques’ various drawbacks limit their application in complex environments [36]. SPR sensors can provide higher analytical accuracy with a smaller sample amount [37].

Sensor surface regeneration plays a key role in reducing fiber-optic SPR sensors’ cost [38,39]. The regeneration process involves selecting an appropriate acidic or alkaline solution to break the chemical bond between the antibody and the target analyte antigen to dissociate the antibody from the fiber-optic sensing region’s surface while maintaining the sensor’s reusability [40]. Different solutions’ abilities to elute analytes vary. Ligand–analyte dissociation effects may differ when different regeneration solutions are used for a particular analyte so that efficient dissociation of that analyte can be realized; however, care must be taken to prevent the regeneration treatment solution from damaging the connected ligand. In this study, the elution abilities of Enro and Cip antibiotics and their respective antibodies were investigated using common solutions such as hydrochloric acid, sodium hydroxide, and glycine–hydrochloric acid solutions, and the regeneration solutions’ concentrations were optimized.

**Figure 1:**
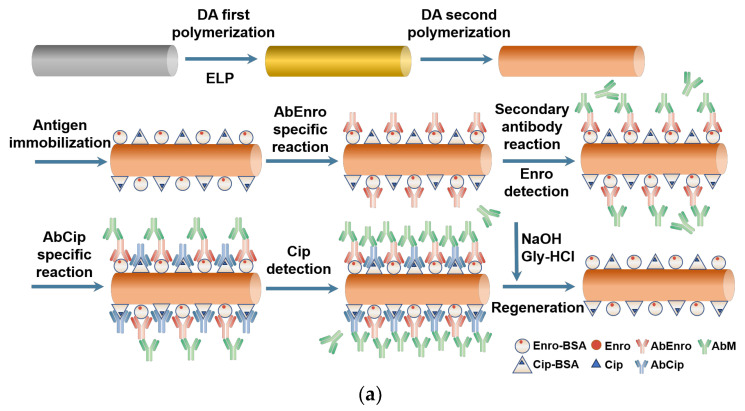

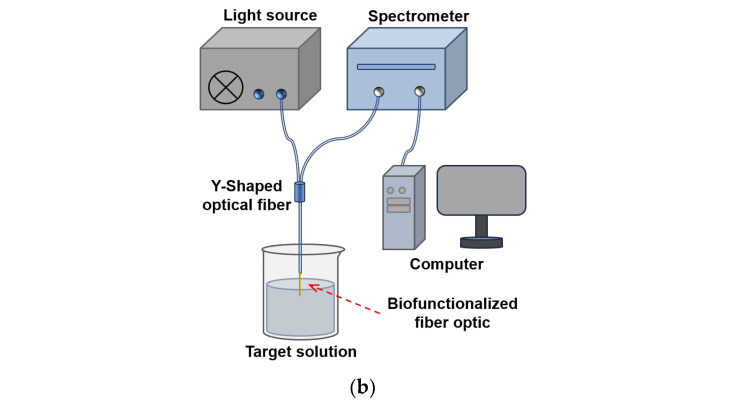
(**a**) The enrofloxacin/ciprofloxacin detection procedure and fiber-optic SPR sensor regeneration. (**b**) Schematic diagram of the SPR detection device.

Our previous studies developed the electroless plating (ELP) approach for preparing fiber-optic SPR sensors [41,42]. This study focuses on the detection of two common fluoroquinolone antibiotics, Enro and Cip, using fiber-optic SPR sensors. A new approach for the sequential detection of two antibiotics was developed by attaching both Enro and Cip to a single optical fiber, which enabled concurrent detection of these two antibiotics (Figure 1). In addition, the sequential detection of Enro and Cip in milk samples was performed and the sensor’s regeneration solution was screened.

## 2. Materials and Methods

### 2.1. Materials

Glycine (Gly), dopamine, enrofloxacin (Enro), ciprofloxacin (Cip), and chlortetracycline were purchased from Shanghai Aladdin Biochemical Science and Technology Co., Ltd. (Shanghai, China). Optical fibers were purchased from Ocean Optics (Shanghai) Co., Ltd. (Shanghai, China). BSA-conjugated enrofloxacin (Enro-BSA), BSA-conjugated ciprofloxacin (Cip-BSA), mouse anti-enrofloxacin antibody (AbEnro), mouse anti-ciprofloxacin antibody (AbCip), and sheep anti-mouse secondary antibody (AbM) were purchased from Shanghai Zhuoshi Biotechnology Co. (Shanghai, China). The actual samples were a milk brand purchased from supermarkets.

### 2.2. Dopamine Functionalization Modification of Fiber-Optic SPR Sensors

Fiber-optic SPR sensors were prepared as described in our previous studies [35,36]. Fiber-optic SPR sensors were reacted with a 2 mg/mL dopamine-Tris solution. The reaction was shaken at 120 r/min in a 25 °C environment for 20, 30, 40, 50, and 60 min. The optical fiber was detected using a spectrometer, and data such as half peak width and peak value were recorded. The optical fiber was reacted with 20 μg/mL of Enro-BSA solution or Cip-BSA solution at 37 °C for 90 min, and active sites on the optical fiber’s surface were closed using ethanolamine (1 M, pH 8.5).

### 2.3. Biofunctionalization Modification and Detection of Fiber-Optic SPR Sensor

The sandwich method was used for individual detection. First, 1 μg/mL of enrofloxacin solution was prepared. Next, 20 μg/mL of enrofloxacin antibody (AbEnro) was incubated with different Enro concentrations (0, 10, 20, 30, 40, 50, 75, 100, and 150 ng/mL) for 2 min to obtain the AbEnro-Enro mixture. Next, the fiber-optic sensor prepared in Section 2.2 with attached Enro-BSA was immersed in the AbEnro-Enro mixture for 30 min, and then the fiber was reacted with 20 μg/mL of secondary antibody solution (AbM) for 30 min. The resonance wavelength’s shift value was recorded during the reaction process. After this reaction, the same steps were performed using Cip and its respective antibody and mixtures.

The sandwich method was also used to sequentially detect Enro and Cip. First, 1 μg/mL solutions of enrofloxacin and ciprofloxacin were prepared. Next, 20 μg/mL of AbEnro was incubated with different Enro concentrations (0, 10, 20, 30, 40, and 50 ng/mL) for 2 min to obtain the AbEnro-Enro mixture. The fiber-optic sensor prepared in Section 2.2 with attached Enro-BSA was immersed in the AbEnro-Enro mixture for 30 min, and then the fiber was reacted with 20 μg/mL of AbM for 30 min. At the same time, 20 μg/mL of AbCip was mixed with different Cip concentrations (0, 10, 20, 30, 40, and 50 ng/mL) and incubated for 2 min. After the reaction between the sensor and AbM, the sensor continued to react with the AbCip-Cip mixture for 30 min. The fiber was then reacted with 20 μg/mL of AbM for 30 min. During the reaction, a spectrometer was used to monitor and record SPR signal profile changes in real time.

### 2.4. Milk Sample Additive Recovery Test

Milk samples were processed as follows:

(1) Enrofloxacin was added to separate milk samples to reach concentrations of 10, 20, 30, 40, and 50 ng/mL; ciprofloxacin was added to separate milk samples to reach concentrations of 10, 20, 30, 40, and 50 ng/mL;
(2) Each milk samples’ pH was adjusted to 4.6, followed by centrifugation. The supernatant was collected, its pH was adjusted to 7.0, and it was subjected to a second round of centrifugation;
(3) Each supernatant’s upper layer was collected and centrifuged for 15 min using a 3000 molecular weight cutoff ultrafiltration tube. The resultant filtrate was stored at 4 °C for future use.

Next, 20 μg/mL of AbEnro was incubated with milk supernatant containing added antibiotics for 2 min, followed by the addition of 20 μg/mL of AbM. A fiber-optic sensor precoated with Enro-BSA and Cip-BSA was then inserted into the solution for 30 min, and the SPR signal curve was recorded using a spectrometer. Another milk supernatant was also mixed with 20 μg/mL of AbCip and incubated for 2 min, after which 20 μg/mL of AbM was added. The fiber-optic sensor was then inserted into this solution for 30 min, and the SPR signal curve was recorded using a spectrometer.

### 2.5. Screening of Regeneration Fluids

Regeneration is a critical process that enables the sensor’s reuse, which increases detection efficiency. To obtain optimal results, the optical fiber’s baseline and sample adsorption should not change after regeneration. Ideally, the analyte and fiber-optic sensing region can be well dissociated to bring the sensing region back to a usable state during regeneration. After 0.1 M hydrochloric acid, 0.01 M hydrochloric acid, 50 mM NaOH, 20 mM NaOH, and 10 mM Gly solutions were prepared, the Gly and HCl solutions’ pHs were adjusted to 1.2 and 2.0, respectively. After antibody detection, the fiber-optic sensing area was immersed in the regeneration solution for 1 min and then rinsed with PBS solution. The SPR signal was tested using a spectrometer, and all these tests were repeated three times each to minimize errors.

## 3. Results and Discussion

### 3.1. Characterization of Fiber-Optic SPR Sensors for PDA Functionalization

**Figure 2:**
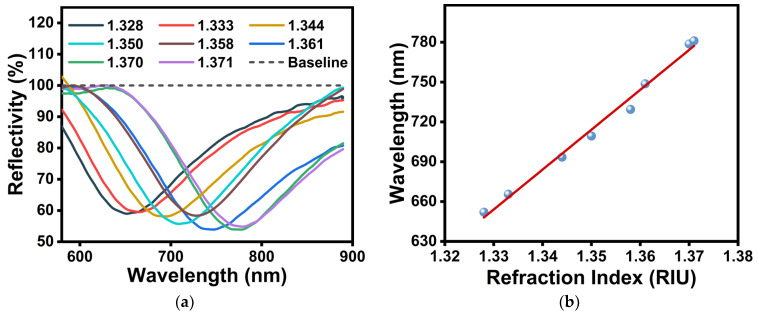
(**a**) Reflectivity spectra of PDA-functionalized fiber-optic SPR sensors in solvents with different refractive indices. Solvents used included water (RI = 1.333), methanol (RI = 1.328), methyl formate (RI = 1.344), diethyl ether (RI = 1.35), pentane (RI = 1.358), ethanol (RI = 1.361), formic acid (RI = 1.370), and acetic acid (RI = 1.371). (**b**) Relationship between wavelength shifts and refractive index changes.

We measured the sensor’s reflectance spectra in varying refractive indices to estimate the polydopamine (PDA)-functionalized fiber-optic SPR sensor’s sensitivity to the surrounding refractive index of the solution. Refractive index sensitivity (RIS) is defined as *S* = ΔSPR/Δ*n*, where Δ*n* is the change in the solution’s refractive index and ΔSPR is the shift in the corresponding resonance wavelength. As shown in Figure 2, the sensor used has a high sensitivity of approximately 2900 nm/RIU, which is higher than or close to other sensors’ sensitivity (see Table S1 for details). In addition, full width at half maximum (FWHM) (defined as the width of the curve corresponding to half of the maximum peak intensity) was used to evaluate the sensor’s resolution capability. The fiber-optic sensor’s FWHM values in different solutions with refractive indices ranging from 1.328 to 1.371 were between 128.6 nm and 155.32 nm. As the refractive index rose, the FWHM value increased, which was consistent with results mentioned in the literature [43,44].

**Figure 3:**
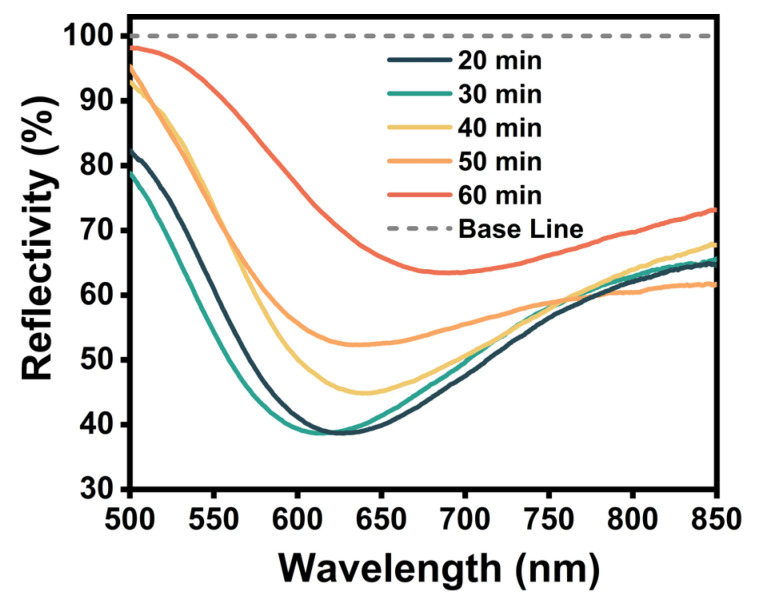
The sensor’s reflection spectra at different PDA reaction times.

Generally, the degree of polymerization and the nature of the PDA layer were affected by the reaction time. As shown in Figure 3, increasing the reaction time from 20 to 30 min did not significantly change the FWHM value (146.6 nm and 149.3 nm, respectively). Extending the reaction time to 50 min significantly increased the FWHM value from 146.6 nm to 199.2 nm. This may be attributed to PDA agglomeration during the longer polymerization time, which led to the formation of large PDA particles that were attached to the sensor surface, which resulted in a notable increase in the half-peak width. Consequently, we established an optimal PDA reaction time of 40 min.

### 3.2. Individual Detection

**Figure 4:**
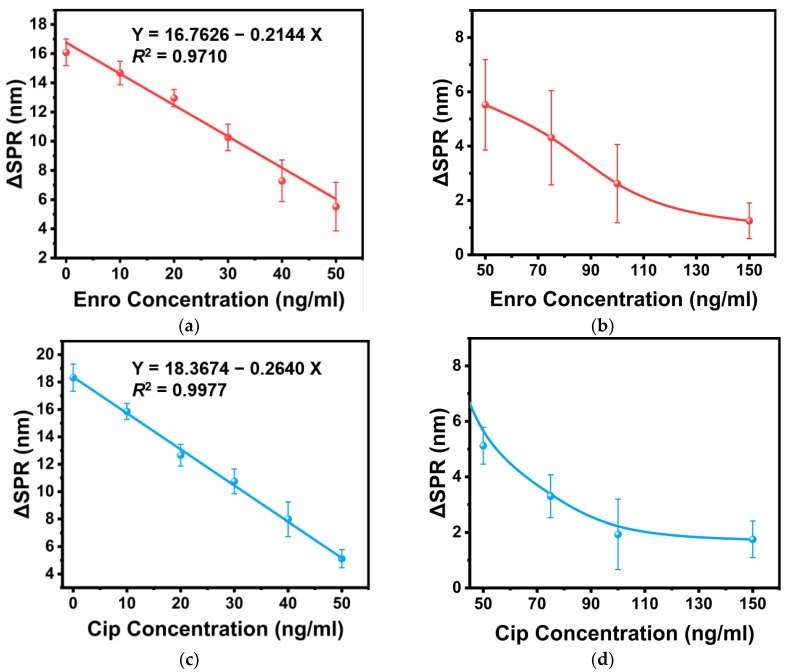
The fiber-optic SPR sensor detects antibiotics in isolation. (**a**) Standard curve for Enro concentrations ranging from 0 to 50 ng/mL. (**b**) Standard curve for Enro concentrations ranging from 50 to 150 ng/mL. (**c**) Standard curve for Cip concentrations ranging from 0 to 50 ng/mL. (**d**) Standard curve for Cip concentrations ranging from 50 to 150 ng/mL. X represents Enro or Cip concentrations, and Y represents peak offset values.

Highly sensitive SPR sensors are utilized to detect molecular interactions. Based on the principle of using antibody-antigen-specific immunity to detect antibiotics, we had to detect Enro and Cip separately. To ensure that antibodies efficiently bound to their respective antigens, a certain amount of AbEnro was mixed and incubated using different concentrations of Enro. We found that the resonance wavelength shift (ΔSPR) gradually decreased as the Enro concentration increased (0–150 ng/mL) because the sandwich method detected the incubation solution’s AbEnro content that had not reacted with Enro. The solution’s AbEnro content was fixed at the beginning, and some AbEnro interacted with Enro during the incubation process. As the solution’s Enro content increased, the free AbEnro content decreased. When AbM was injected into the system, it specifically bound to AbEnro’s surface, which achieved the effect of expanding the signal response value. As shown in Figure 4a, the curve had a good linear relationship when the enrofloxacin concentration was 0–50 ng/mL, and a 2.01 ng/mL limit of detection (LOD) was obtained. The LOD was defined as the concentration at which the signal was equal to three times the standard deviation of the signal from the measurement blank solution. As shown in Figure 4b, when the Enro concentration exceeded 50 ng/mL, the relationship between ΔSPR and Enro concentration was no longer linear. It can be inferred that the binding of Enro to AbEnro had gradually reached saturation at this time, so the magnitude of the change in ΔSPR was no longer significant, even if the Enro concentration continued to increase. A similar operation was performed for the Cip assay, as shown in Figure 4c,d. Similarly, the ΔSPR signal value gradually decreased as the Cip concentration increased from 0 to 150 ng/mL. The curve had good linearity when the Cip concentration was 0–50 ng/mL, and its LOD was 1.17 ng/mL.

### 3.3. Joint Detection

**Figure 5:**
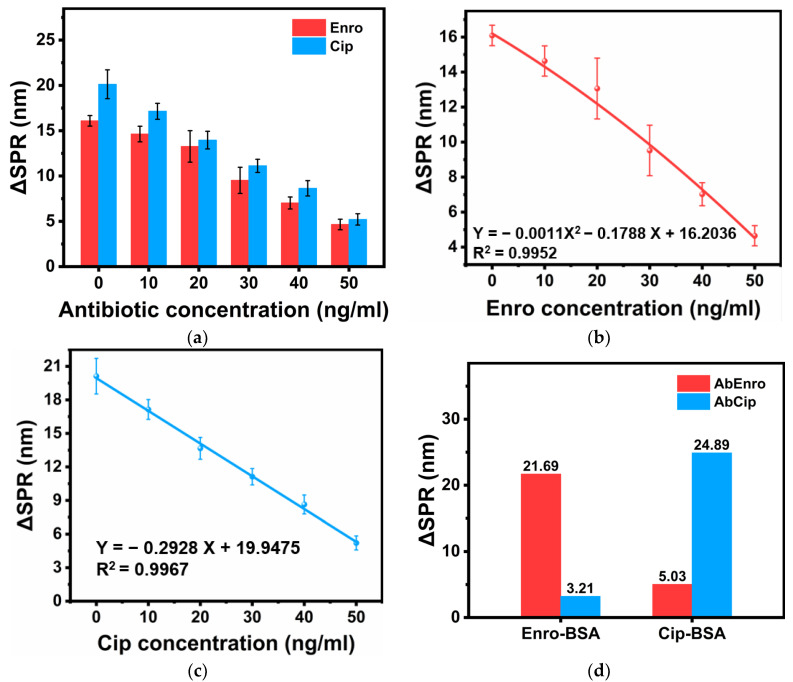
(**a**) Fiber-optic SPR spectra for monitoring sample response shifts based on AbM’s response shift. (**b**) Standard curve for enrofloxacin detection. (**c**) Standard curve for ciprofloxacin detection. (**d**) Cross-reactivity results between two antigens (Enro-BSA and Cip-BSA) and two antibodies (AbEnro and AbCip). X represents Enro or Cip concentrations, and Y represents peak offset values.

The same fiber-optic SPR sensor was used for Enro and Cip joint detection. To ensure accurate results and prevent crosstalk effects between antibodies, it was necessary to perform an anti-interference test between Enro and Cip. As shown in Figure 5d, no large fluctuations in signal values were observed when the sensor connected to Enro-BSA was immersed in the AbCip solution or when the sensor connected to Cip-BSA was immersed in the AbEnro solution, indicating that the antibody-antigen binding was specific. Cip’s ΔSPR was obtained by subtracting Enro’s ΔSPR from the total signal response value. As Enro and Cip concentrations changed, their respective signal values changed. Enro’s and Cip’s corresponding standard curves are plotted separately in Figure 5b,c, which show that both antibiotics’ fitting results were relatively satisfactory, with respective LODs of 0.81 ng/mL and 1.20 ng/mL. This type of combined detection can significantly increase fiber-optic sensors’ versatility and give full play to their miniaturization and portability.

### 3.4. Adding Recycling Tests to Practical Systems

To verify the solution competition method’s effectiveness in actual samples, it was necessary to pre-add different concentrations of antibiotics to milk and then perform recovery tests to detect Enro and Cip in milk. Before the SPR test, we had to remove complex proteins and fats from the milk, as they can produce some non-specific adsorption. We performed several ultrafiltrations and centrifugations to reduce the effect of these interfering factors after adding Enro and Cip. According to the SPR principle, wavelength shifts are related to changes in the sensor surface’s refractive index, which are attributed to the binding or adsorption of the antibodies onto the sensor surface. That is, as the antibiotic concentration in a sample increases, less antibodies are bound to the sensor surface, and thus the ΔSPR value decreases. Therefore, in the joint detection process using milk samples, according to the relationship between ΔSPR and Enro and Cip concentrations obtained from Figure 5, we could calculate the corresponding antibiotic concentrations using the measured ΔSPR values. Dividing the calculated results by the actual added content allowed us to compute the recovery rate. As shown in Table 1, the recovery rates of Enro and Cip in milk were 96.46–120.46% and 96.74–126.90%, respectively. Some deviations between the samples’ recoveries and the actual spiked amounts were observed, which might have been due to the influence of instrumental noise, deviation in the standard curve fitting, and matrix effects that may have affected the results. In any case, the final results were within a reasonable range and did not affect the application in trace sample testing. In addition, we compared this study’s results from Enro and Cip assays on actual samples with previously reported results, as shown in Table 2. Compared with other methods, our approach using fiber-optic SPR sensors for detecting trace amounts of antibiotics achieved top-tier levels in terms of detection concentration range, recovery rate, and accuracy.

### 3.5. Recycled Liquid Screening

**Figure 6:**
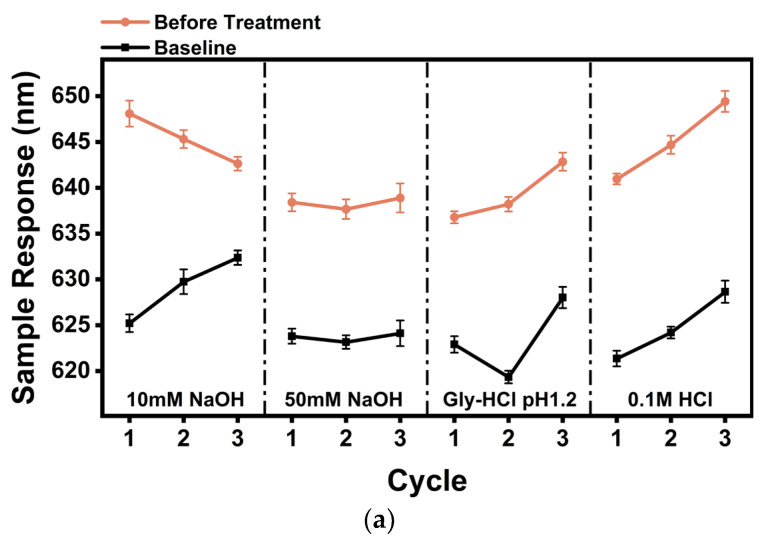

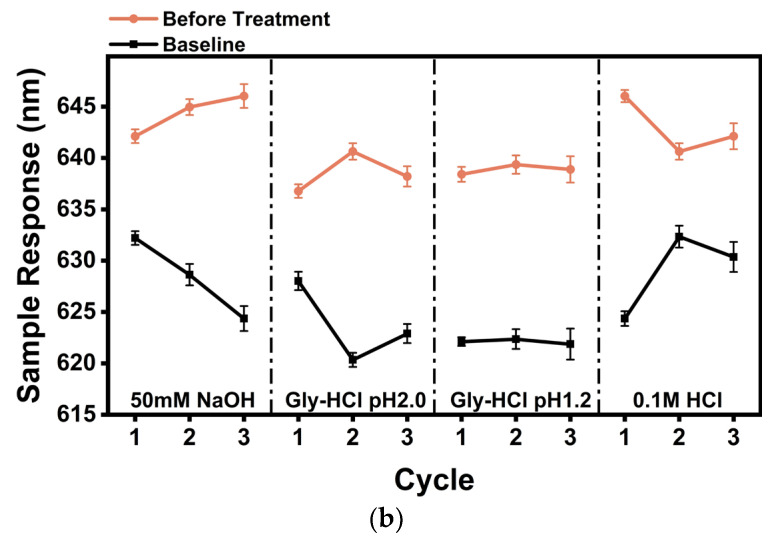
Comparison of baseline shifts and sample responses using four regeneration solutions for (**a**) AbEnro and (**b**) AbCip. Circles (orange) and squares (black) represent the sample responses and baselines, respectively. Three cycles of each regeneration solution were tested, and are separated by dashed lines to compare stability after regeneration using these solutions.

The ability of different solutions to elute analytes varies. For a particular analyte, the dissociation effect of the ligand–analyte can be completely distinct when using different regeneration solutions. Consequently, efficient dissociation of a particular analyte can be realized. Meanwhile, care needs to be taken to prevent the regeneration treatment solution from damaging the connected ligand. Three cycles of each regeneration solution were tested to compare stability after regeneration. As shown in Figure 6a, we comparatively analyzed the baseline shifts and sample response values for AbEnro elution using four regeneration solutions, namely, 0.1 M HCl, Gly-HCl (pH 1.2), 10 mM NaOH, and 50 mM NaOH. Baseline and resonance wavelength shift values remained stable without significant changes when using the 50 mM NaOH regeneration solution. However, baseline values gradually increased when using 0.1 M HCl and 10 mM NaOH regeneration solutions, indicating that AbEnro was not completely eluted from the surface of the optical fiber. A larger baseline value fluctuation when using the Gly regeneration solution also indicated that a small amount of Enro remained bound to the optical fiber’s surface and was not completely eluted. Therefore, the 50 mM NaOH solution was the best solution for Enro elution compared with other regeneration solutions. Similar to the AbEnro regeneration described above, AbCip was eluted using 0.1 M HCl, pH 1.2 Gly-HCl, pH 2.0 Gly-HCl, and 50 mM NaOH. Figure 6b illustrates that the sample baseline and response values showed large fluctuations after regeneration using NaOH, 0.1 M HCl, and pH 2.0 Gly-HCl solutions, indicating that the antibody on the optical fiber could not be completely eluted and that it was not suitable for AbCip elution. Surprisingly, only pH 1.2 Gly-HCl could be maintained to achieve results that are more satisfactory. Therefore, pH 1.2 Gly-HCl solution was more suitable for AbCip elution.

## 4. Conclusions

In summary, we developed a new method to detect Enro and Cip sequentially using a portable fiber-optic SPR sensor. The fiber-optic sensor’s sensitivity was 2900 nm/RIU, and Enro and Cip LODs were calculated to be 1.20 ng/mL and 0.81 ng/mL, respectively. Furthermore, recovery studies of Enro and Cip implemented in milk samples were satisfactory. Compared with other results in the literature, the sensor prepared in this study exhibited good sensitivity, detection limit, and recovery rate performances when detecting antibiotic samples. In addition, sodium hydroxide and glycine-HCl were demonstrated as efficient regenerating solutions for AbEnro and AbCip elution, respectively, which facilitated the sensor’s reuse. We believe that the fiber-optic SPR assay proposed in this paper has significant potential in food safety testing applications.

## Figures and Tables

**Table 1:** Recovery study of different concentrations of Enro and Cip in milk samples.

Target Sample	Spiked Concentration (ng/mL)	Wavelength Shift (nm)	Average ± SD (ng/mL)	Recovery (%)
Enro	0	16.01 ± 0.17	1.076 ± 0.93	/
	10	13.89 ± 0.05	12.04 ± 0.24	120.46
	20	11.57 ± 0.09	22.73 ± 0.39	113.66
	30	9.29 ± 0.08	32.26 ± 0.32	107.53
	40	7.62 ± 0.11	38.76 ± 0.42	96.90
	50	5.02 ± 0.06	48.23 ± 0.51	96.46
Cip	0	19.01 ± 0.11	3.21 ± 0.37	/
	10	16.23 ± 0.17	12.69 ± 0.59	126.90
	20	12.96 ± 0.13	23.87 ± 0.44	119.35
	30	10.25 ± 0.16	33.13 ± 0.50	110.43
	40	7.56 ± 0.18	42.30 ± 0.62	105.75
	50	5.78 ± 0.14	48.37 ± 0.50	96.74

**Table 2:** Comparative results with other recoveries reported in the literature.

Target Analytes	Detection Methods	Detection Range	LOD	Recovery (%)	Refs.
Enro	Fluorescent probe	63–60,000 ng/mL	8 ng/mL	83.7–87.7	[45]
Enro/Cip	Fluorescent probe	1–100 ng/mL	0.061 ng/mL (Enro) 0.053 ng/mL (Cip)	80.1–102.48	[9]
Enro/Cip	LC		48 ng/mL (Enro) 2 ng/mL (Cip)	79 (Enro) 88 (Cip)	[46]
Enro/Cip	Molecularly imprinted polymers	0.2–5 ng/mL	16 ng/mL (Enro) 19 ng/mL (Cip)	82.6–93.5 (Enro) 81.2–94.8 (Cip)	[47]
Enro	Colloidal gold-immunochromatographic sensor	0.25–1 ng/mL	0.13 ng/mL	88.9–108.5	[48]
Enro	Eu-fluorescence-immunochromatographic sensor	0.25–1 ng/mL	2.5 ng/mL	88.6–113.6	[48]
Enro	Elisa	10–30 ng/mL	0.7 ng/mL	72.9–113.36	[49]
Enro	Molecularly imprinted polymers	2.8–28,000 pM	0.9 pM	96.4–102	[50]
Enro	Polymer-grafted nanographite	0.1–60 μg/mL	8.5 ng/mL	68.5–81.2	[51]
Enro/Cip	Fiber-optic SPR sensor	0–50 ng/mL	0.81 ng/mL (Enro) 1.20 ng/mL (Cip)	96.46–120.46 (Enro) 96.74–126.9 (Cip)	This study

## Data Availability

Data are available upon reasonable request from the corresponding author.

## Supplementary Materials

The following supporting information can be downloaded at: https://www.mdpi.com/article/10.3390/s24072126/s1, Table S1: Comparison of the sensitivity of different types of SPR sensors. References [52,53,54,55,56] are cited in the Supplementary Materials.
