# Supplementary material for: Efficient Sequential Detection of Two Antibiotics Using a Fiber-Optic Surface Plasmon Resonance Sensor
Source: Sensors (Basel). 2024 Mar 26;24(7):2126. doi: 10.3390/s24072126 (PMC11013968; doi:10.3390/s24072126)
Supplement: Supplementary file 1 [file sensors-24-02126-s001.zip › sensors-2886503-supplementary.pdf]

Supplementary Materials for

Efficient Sequential Detection of Two Antibiotics Using a Fiber-Optic Surface Plasmon Resonance Sensor

Ze Zhao [1,†], Huiting Yin [2,†], Jingzhe Xiao [1], Mei Cui [1], Renliang Huang [3,*] and Rongxin Su [1,2,*]

1. State Key Laboratory of Chemical Engineering, Tianjin Key Laboratory of Membrane Science and Desalination Technology, School of Chemical Engineering and Technology, Tianjin University, Tianjin 300072, China; zhao_ze@tju.edu.cn (Z.Z.); jingzhexiao@tju.edu.cn (J.X.); meicui@tju.edu.cn (M.C.)
2. Zhejiang Institute, Tianjin University, Ningbo 315201, China; yinht97@tju.edu.cn
3. Tianjin Key Laboratory for Marine Environmental Research and Service, School of Marine Science and Technology, Tianjin University, Tianjin 300072, China

* Correspondence: tjuhrl@tju.edu.cn (R.H.); surx@tju.edu.cn (R.S.)

† These authors contributed equally to this work.

Table S1. Comparison of the sensitivity of different types of SPR sensors

Type of Sensor	Refractive Index Linear Range (RIU)	Sensitivity (nm/RIU)	Refs.
D-shaped SPR sensor with MXene	1.333-1.354	3143	[52]
Two-dimensional nanomaterial violet phosphorus SPR sensor	1.33-1.34	2335.64	[53]
Side-polish plastic optical fiber	1.335-1.39	2008.58	[54]
Fiber cladding SPR sensor based on V-grooves structure	1.333-1.385	1862.33	[55]
Sputtering gold film SPR sensor	1.333-1.347	1557	[56]
Fiber optic PDA functionalized fiber optic SPR sensor	1.328-1.371	2900	This work

References

52. Zhou, Y.; Yan, X. D-Shaped Fiber Surface Plasmon Resonance Refractive Index Sensor Enhanced By MXene (Ti3C2Tx). *IEEE Photonics Journal* **2022**, 14, 7151607.
53. Jing, J.-Y.; Liu, K.; Wu, Z.; Liu, Y.-M.; Jiang, J.-F.; Xu, T.-H.; Yan, W.-C.; Xiong, Y.-Y.; Zhan, X.-H.; Xiao, L.; et al. Violet phosphorus-enhanced plug-and-play double-lane fiber optic surface plasmon resonance refractometer. *Acta Physica Sinica* **2023,** 72, 214206.
54. Teng, C.; Ying, S.; Min, R.; Deng, S.; Deng, H.; Chen, M.; Chu, X.; Yuan, L.; Cheng, Y.; Xue, M. Side-Polish Plastic Optical Fiber Based SPR Sensor for Refractive Index and Liquid-Level Sensing. *Sensors* **2022**, 22, 6241.
55. Li, L.; Wei, Y.; Tan, W.; Zhang, Y.; Liu, C.; Ran, Z.; Tang, Y.; Su, Y.; Liu, Z.; Zhang, Y. Fiber cladding SPR sensor based on V-groove structure. *Optics Communications* **2023**, 526, 128944.
56. Suzuki, H.; Sugimoto, M.; Matsui, Y.; Kondoh, J. Effects of gold film thickness on spectrum profile and sensitivity of a multimode-optical-fiber SPR sensor. *Sensors and Actuators B-Chemical* **2008**, 132, 26-33.
